# Development of a Thai tool for assessing behavioral and psychological symptoms of dementia: A confirmatory factor analysis

**DOI:** 10.1002/brb3.1816

**Published:** 2020-08-28

**Authors:** Harisd Phannarus, Weerasak Muangpaisan, Pitiporn Siritipakorn, Sudarat Pianchob, Orawan Supapueng

**Affiliations:** ^1^ Department of Preventive and Social Medicine Faculty of Medicine Siriraj Hospital Mahidol University Bangkok Thailand; ^2^ Department of Nursing Faculty of Medicine Siriraj Hospital Mahidol University Bangkok Thailand; ^3^ Department of Mental Health and Psychiatric Nursing Faculty of Nursing Mahidol University Bangkok Thailand; ^4^ Division of Clinical Epidemiology Department of Research Faculty of Medicine Siriraj Hospital Mahidol University Bangkok Thailand

**Keywords:** behavioral and psychological symptoms of dementia, caregiver burden, confirmatory factor analysis, dementia, europsychiatric Inventory

## Abstract

**Introduction:**

The early recognition and management of the behavioral and psychological symptoms of dementia (BPSD) are important to inform treatment decisions. Current BPSD screening tools are time‐consuming and require advanced skills, limiting their application in routine clinical practice. An easier and quicker tool for use by nonphysician healthcare personnel is needed.

**Methods:**

A 14‐item, Thai‐language, BPSD scoring system for dementia (BPSD‐T) was developed, based on clinical surveys and modifications after a pilot study. The Neuropsychiatric Inventory (NPI), BPSD‐T, Thai Mental State Examination (TMSE), Clinical Dementia Rating Scale (CDR), and Barthel Index were performed. BPSD‐T and NPI scores were compared, and test validity and reliability were analyzed.

**Results:**

A total of 168 people with dementia (mean age, 80.7 ± 6.7 years) and their primary caregivers were recruited. A total of 105 (62.5%) subjects were diagnosed with Alzheimer's disease (AD), and 31 (18.5%) with AD with small‐vessel disease. The Global CDR was 0.5–1 for 73.8% of subjects, and 2–3 for 26.2%. The BPSD‐T content validity index was 0.80–0.98, with high inter‐rater and test–retest reliability. Confirmatory factor analysis showed the goodness of fit of 5 clusters of BPSD‐T included a psychomotor syndrome (aggression, irritability, delusions, insomnia), an affective syndrome (apathy, repeating, anxiety, depression), a psychosis syndrome (misidentification, hallucinations), a behavior syndrome (hoarding, rummaging, wandering), and a euphoria syndrome (euphoria). Convergent validity showed a high correlation of the frequency score (*r* = 0.66) and caregiver distress score (*r* = 0.76) with the NPI. The BPSD‐T score was significantly higher with more severe dementia. The average completion time for the BPSD‐T (230.9 ± 65.5 s) was significantly less than that for NPI (506 ± 196.9 s; *p* < .001).

**Conclusions:**

BPSD‐T is a quick, reliable, and valid test to evaluate BPSD from the common dementia subtypes and severity, with a good correlation with the NPI. Its application in routine clinical practice will enable earlier recognition, targeted intervention, improved quality of care, and reduced caregiver burden.

## Graphical abstract

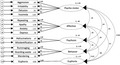

## 1 INTRODUCTION

Globally, the human population is rapidly aging due to improvements in healthcare education and sanitation, better treatments, and advanced facilities. Dementia is a common condition in older adults. Currently, there are an estimated 25 million patients worldwide, and up to 50 million additional cases are expected over the next 20 years (Qiu, Kivipelto, & von Strauss, 2009). There are many symptoms of dementia, including global cognitive decline sufficient to affect functional independent activities and neuropsychiatric symptoms, collectively referred to as behavioral and psychological symptoms of dementia (BPSD). BPSD is present in 60%–90.5% of people with dementia (PwD) (Ferri, Ames, & Prince, 2004; Selbaek, Kirkevold, & Engedal, 2008). The occurrence of BPSD waxes and wanes, and it is found in every stage of the disease (Lyketsos et al., 2002). BPSD is comprised of psychotic symptoms, mood symptoms, sleep symptoms, and other phenomena. Clustering of the BPSD occurs in PwD (Aalten et al., 2007; Srikanth, Nagaraja, & Ratnavalli, 2005). The study of a cluster of BPSD instead of exploring each BPSD separately may reveal an underlying neurobiological pathogenesis which might relate to clinical practice and become a target of intervention trials. A factor analysis has shown that the factor structure of BPSD is dependent on dementia severity (Aalten et al., 2007, 2008; Poletti, Nuti, Cipriani, & Bonuccelli, 2013). For example, psychosis frequently coexisted with agitated behaviors as dementia progressed (Aalten et al., 2008). BPSD has serious consequences in terms of worsening disability, increased caregiver (CG) burden, and earlier institutionalization (Lawlor & Bhriain, 2001; Lyketsos et al., 2002; Machnicki, Allegri, Dillon, Serrano, & Taragano, 2009; de Vugt et al., 2005).

There are several clinical tests that have high validity and can reliably evaluate BPSD, such as the Neuropsychiatric Inventory (NPI) (Cummings, 1997) and the Behavioral Pathology in Alzheimer's Disease (BEHAVE‐AD) Rating Scale (Reisberg, Auer, & Monteiro, 1996). The NPI is a proven instrument in PwD and assesses all types of dementia and stages of the disease. Four subsyndromes of NPI—namely, hyperactive behaviors, psychosis, affective behaviors, and apathy—have consistently been found in studies via factor analysis (Aalten et al., 2008). However, the analysis did not include non‐NPI symptoms and mostly used exploratory factor analysis to explore the factor components. There are some limitations of exploratory factor analysis, including its high sensitivity to the variables being subjected to the analysis and the chance of correlations with other relevant BPSD symptoms. Confirmatory factor analysis (CFA) tests the BPSD grouped priori based on theoretical or clinically meaningful entities. Therefore, the cluster of BPSD from the CFA is likely to make more clinical sense.

The features and rates of BPSD have varied immensely across different ethnic groups, depending on the methodology, setting, type and severity of the disease, and cultural factors (Shah, Dalvi, & Thompson, 2005). Several possible causes might explain the variation by cultural factor, such as differing perceptions of BPSD, differing healthcare‐seeking practices by the CGs of PwD, and differing availability and systems of care services (Cohen, Hyland, & Magai, 1998; Shah et al., 2005). Apathy and other negative symptoms, such as depression, are more common in PwD and result in a heavy CG burden in western cultures (Fuh, Lam, Hirono, Senanarong, & Cummings, 2006; Prince, 2009). In Eastern cultures, symptoms such as aggression, aberrant motor behavior, disinhibition, and irritability are the most common and worsen the CG burden, especially in Thai society (Charernboon & Phanasathit, 2014; Muangpaisan et al., 2010; Pinidbunjerdkool, Saengwanitch, & Sithinamsuwan, 2014; Senanarong et al., 2005; Taemeeyapradit, Udomittipong, & Tepparak, 2014).

While established BPSD assessment tools are widely used in research, they are impractical for the fast pace of routine clinical practice in outpatient settings. Traditional tests require 10–20 min to conduct and need an experienced assessor (Drachman, Swearer, O’Donnell, Mitchell, & Maloon, 1992; Kang et al., 2004; Monteiro et al., 2001). There is a need for a quick and accurate/reliable instrument for use in outpatient settings by nonphysician healthcare personnel. Therefore, we aimed to develop a new instrument for primary CGs who are familiar with their PwD's behavior. The tool was based on the most common and burdensome symptoms reported by community‐dwelling BPSD surveys in Thailand.

## 2 METHODS

### 2.1 Subjects and ethics approval

This cross‐sectional study was conducted between April 2018 and February 2019. All subjects or their legal representatives provided written informed consent. All participants had received a diagnosis of any type of dementia according to the Diagnostic and Statistical Manual of Mental Disorders (DSM‐5) criteria, without regard to severity. (*American Psychiatric Association. Diagnostic and Statistical Manual of Mental Disorders, 5th Ed. Arlington:*, 2013, n.d.) A primary CG who had taken care of the PwD for at least 4 hr/day and 4 days/week was present during the assessment (Cummings, 1997). Subjects with other psychological diseases, including delirium, or whose primary CG could not communicate with the assessor, were excluded. The subjects were randomly selected using a systemic sampling method that generated a sequence from the outpatient queue numbers of PwD at the Geriatric Clinic at Siriraj Hospital, a major university hospital in Thailand.

### 2.2 Instrumental development

The authors systematically searched electronic databases to identify previous BPSD studies in the Thai community‐dwelling dementia population (Charernboon & Phanasathit, 2014; Graipaspong, Thaipisuttikul, & Vallipakorn, 2016; Muangpaisan et al., 2010; Pinidbunjerdkool et al., 2014; Senanarong et al., 2005; Taemeeyapradit et al., 2014). These studies mostly reported on the prevalence of BPSD and CG burden. After reviewing the Thai and international studies and the construct conceptualization was specified, the authors selected 20 items as the most severe and troublesome BPSD to include in a new instrument, referred to as the Behavioral and Psychological Symptom of Dementia assessment tool, Thai version (BPSD‐T). The BPSD‐T was assessed for content validity index by two geriatric psychiatrists, two geriatricians, and one neurologist (LAWSHE, 1975). After a pilot study, some items were adjusted to improve comprehensibility.

The BPSD‐T was scaled as “presence” or “absence” of symptoms during the past month. If the primary CG confirmed that the PwD had a specific symptom, the assessor then asked two additional questions focusing on symptom frequency and CG distress. The frequency of symptoms of each item was scaled 1–4 (1 = less than 2 times/month; 2 = 2–3 times/month; 3 = weekly or at least 4 times/month; 4 = almost every day). The CG distress score was rated on a 4‐point scale (1 = no effect on CGs; 2 = little effect; 3 = some effects but still bearable; 4 = a lot of effect which cannot be handled). An English translation of the BPSD‐T is shown in Supplementary Data S1.

### 2.3 Data collection

The investigators collected demographic data of all PwD and CG. The NPI was employed as the standard BPSD assessment, with its results reviewed by a senior consultant in geriatric neurology. The NPI is widely used to assess neuropsychiatric disturbances through interviews with the primary CG. It encompasses 12 behavioral domains, each with a screening question to determine the frequency and severity of a symptom, and a CG distress rating scale. The Thai Mental State Examination (TMSE) (“Train the Brain Forum Committee. Thai Mental State Examination (TMSE),” 1993), a translated and culturally modified version of the Mini‐Mental State Examination (MMSE) (Folstein, Folstein, & McHugh, 1975), was also completed. Disease severity was assessed using the Clinical Dementia Rating (CDR) Scale (Morris, 1997), and the activities of daily living were assessed using the Barthel Index (Laohaprasitiporn, Jarusriwanna, & Unnanuntana, 2017). The BPSD‐T was performed at the time of recruitment by two independent assessors who were blinded to the results of the NPI. The BPSD‐T was retested 2 weeks later, and the duration of administration was recorded. All tests were completed for each subject in less than 30 min.

### 2.4 Statistical analysis

SPSS version 18.0 was used for statistical analysis (SPSS Inc., PASW Statistics for Windows, Chicago, Illinois). Demographic data of the participants were analyzed using descriptive statistics, that is, percentage, mean, and standard deviation.

The frequency component of a cluster of BPSD‐T was used in the factor analysis. Before the analysis, items with a frequency of less than 5% were excluded, because such items with low frequency would have little variance to contribute to inter‐item correlations (Osborne & Costello, 2004). CFA, using R programming language via lavaan package, was used to examine the proposed nine models of the BPSD‐T and to evaluate each model's goodness of fit. Due to the differences in the items between the BPSD‐T and other tools for BPSD assessment, each factor was grouped into the previous factors in accordance with previous studies of BPSD clusters (Supplementary Data S2).

Model parameter appraisals used maximum‐likelihood (ML) estimation. A chi‐squared test was used to determine goodness of fit. However, several factors affected the chi‐squared test, such as sample size, model size, and the distribution of variables. This could lead to a type I error (unnecessary rejection of the hypothesized model). Therefore, multiple indices—comprising the Comparative Fit Index (CFI), the Tucker–Lewis Index (TLI), Standardized Root‐Mean‐Square Residual (SRMR), and Root‐Mean‐Square Error of Approximation (RMSEA)—were also used to evaluate model fit. The thresholds for these indices for good fit were CFI > 0.90, SRMR < 0.08, and RMSEA < 0.08. By comparison, the thresholds for marginal fit were CFI > 0.87, and SRMR and RMSEA values < 0.10 (Bong, Woo, & Shin, 2013).

Convergent validity was determined by comparing the frequency scores and CG distress scale scores of the BPSD‐T and NPI. Discriminant validity was performed by comparing the BPSD‐T scores per CDR group and TMSE stratification. Internal consistency, including inter‐rater reliability and test–retest reliability, was assessed using the kappa coefficient, which measures the beyond‐chance ratio of the observed agreement to the potential agreement. A kappa of 1 represented complete agreement beyond chance, whereas a kappa beyond 0 indicated agreement at the chance level. Two independent raters scored the BPSD‐T, and their inter‐rater reliability was calculated by correlation analysis. Test–retest reliability was assessed using 32 randomly selected participants (20% of the sample) by conducting a second BPSD‐T interview within 2 weeks of a first assessment by the two independent raters. The study was approved by the Institutional Review Board and the Ethics Committee, Siriraj Hospital, Mahidol University, Thailand (REC 200/2561).

## 3 RESULTS

### 3.1 Characteristics of PwD

One hundred and sixty‐eight PwD were included. Their average age was 80.7 ± 6.7 years, and 117 (69.6%) were female. The participation rate was 86.7%. Ninety‐one subjects (54.1%) were educated at the level of primary school or had no formal education (Table 1). There were 105 cases of Alzheimer's disease (AD), 31 cases of AD with small‐vessel disease (SVD), 23 cases of vascular dementia (VaD), and 9 cases of Parkinson's disease dementia (PDD). The average TMSE score was 17.4 ± 6.5, and the mean duration of dementia diagnosis before the study enrollment was 35.1 months. The Global CDR was 0.5–1 (mild) in 124 (73.8%) subjects, and 2–3 (moderate to severe) in 44 (26.2%) subjects.

**Table 1:** Baseline characteristics of the people with dementia (*n* = 168)

People with dementia	
Age (years), mean ± *SD*	80.7 ± 6.7
Woman, *n* (%)	117 (69.6)
Marital status, *n* (%)	
Widowed	74 (44)
Married	73 (43.5)
Single	15 (8.9)
Education, *n* (%)	
No education	12 (7.1)
Primary school	79 (47)
Secondary school	37 (22)
≥Diploma	40 (23.9)
Health status, *n* (%)	
Hypertension	133 (79.2)
Dyslipidemia	118 (70.2)
Diabetes mellitus	54 (32.1)
Chronic kidney disease	44 (26.2)
Cerebrovascular disease	38 (22.6)
PD (with and without dementia/cognitive impairment)	13 (7.7)
Diagnosis of dementia, *n* (%)	
AD	105 (62.5)
AD with SVD	31 (18.5)
VaD	23 (13.7)
PDD	9 (5.4)
TMSE, mean ± *SD*	17.4 ± 6.5
Global Clinical Dementia Rating Scale, *n* (%)	
0.5	63 (37.5)
1	61 (36.3)
≥2	44 (26.2)
Barthel score, *n* (%)	
100	42 (25)
75–90	76 (45.2)
50–70	23 (13.7)
Duration between diagnosis to enrollment, mean (months)	35.1

Abbreviations: AD, Alzheimer's disease; PD, Parkinson's disease; PDD, Parkinson's disease dementia; *SD*, standard deviation; SVD, small‐vessel disease.

The prevalence of BPSD by NPI was 97%. Table 2 shows the prevalence of each BPSD categorized by CDR and TMSE. The most common BPSD was repeating sentences/activities and insomnia. There was a statistical difference between the BPSD‐T scores of CDR 0.5 and those of CDR 3. Likewise, there was a statistical difference between the BPSD‐T scores of the groups with TMSE scores of 21–30 and 0–10 (Table 2).

**Table 2:** The frequency of each BPSD‐T item, categorized by CDR and TMSE level

Items	CDR				TMSE			Total
	0.5	1	2	3	21–30	11–20	0–10	
Q1: aggression	19%	32.8%	29.6%	35.3%	23.9%	27.9%	34.5%	27.4%
Q2: irritability	31.7%	36.1%	33.3%	29.4%	36.6%	29.4%	34.5%	33.3%
Q3: delusion	7.9%	8.2%	18.5%	5.9%	14.1%	4.4%	10.3%	9.5%
Q4: insomnia	27%	41%	40.7%	58.8%	31%	38.2%	51.7%	37.5%
Q5: apathy	14.3%	27.9%	33.3%	47.1%	18.3%	25%	44.8%	25.6%
Q6: repeating	90.5%	82%	70.4%	70.6%	87.3%	85.3%	62.1%	82.1%
Q7: anxiety	27%	18%	18.5%	17.6%	16.9%	26.5%	20.7%	21.4%
Q8: depression	27%	16.4%	22.2%	29.4%	22.5%	23.5%	20.7%	22.6%
Q9: misidentification	3.2%	8.2%	18.5%	29.4%	0%	11.8%	31%	10.1%
Q10: hallucination	6.3%	19.7%	48.1%	58.8%	7%	25%	58.6%	23.2%
Q11: hoarding	15.9%	26.2%	18.5%	23.5%	16.9%	23.5%	24.1%	20.8%
Q12: rummaging	33.3%	39.3%	25.9%	23.5%	32.4%	38.2%	24.1%	33.3%
Q13: wandering	4.8%	8.2%	3.7%	0%	2.8%	8.8%	3.4%	5.4%
Q14: euphoria	19%	14.8%	22.2%	11.8%	16.9%	14.7%	24.1%	17.3%
Mean frequency score	9.30	11.25	12.30	13.24	9.63	11.18	13.28	10.89
*p* value	0.092^a^	0.092^a^	0.087^b^	0.026*^c^	0.059^g^	0.059^g^	0.016*, ^h^	
	0.087^b^	0.628^d^	0.628^d^	0.262^e^	0.016*, ^h^	0.301^i^	0.301^i^	
	0.026*, ^c^	0.262^e^	0.530^f^	0.530^f^				
	0.077^k^				0.029*^k^			

Abbreviations: BPSD‐T, Thai tool for Assessing Behavioral and Psychological Symptoms of Dementia; CDR, Clinical Dementia Rating; TMSE, Thai Mental State Examination.

^a^ Correlation of BPSD‐T between CDR 0.5 and 1.

^b^ Correlation of BPSD‐T between CDR 0.5 and 2.

^c^ Correlation of BPSD‐T between CDR 0.5 and 3.

^d^ Correlation of BPSD‐T between CDR 1 and 2.

^e^ Correlation of BPSD‐T between CDR 1 and 3.

^f^ Correlation of BPSD‐T between CDR 2 and 3.

^g^ Correlation of BPSD‐T between TMSE 21–30 and 11–20.

^h^ Correlation of BPSD‐T between TMSE 21–30 and 0–10.

^i^ Correlation of BPSD‐T between TMSE 11–20 and 0–10.

^k^ Kruskal–Wallis test.

^*^ P‐value <0.05.

### 3.2 Characteristics of CG

There were 168 CGs with a mean age of 55.9 ± 13.4 years; 136 (81%) were female, and 101 (60.1%) were married. One hundred and seventeen (69.6%) CG were educated to at least a bachelor's degree level, and 160 (95.2%) reported having taken care of the PwD for longer than 1 year (Supplementary Data S3).

### 3.3 Psychometric properties of the BPSD‐T

#### 3.3.1 Content validity

Content validation of the BPSD‐T was determined by five multidisciplinary experts using a content validity index (CVI) of 0.8–0.98 for each item (Table 3).

**Table 3:** Reliability of BPSD‐T

Item	Content validity index, (0–1)	Inter‐rater reliability 168 cases (kappa; 95% CI)	Test–retest reliability 32 cases (Overall percent agreement)
1. Aggression	0.9	0.80 (0.7–0.9)	90.6%
2. Irritability	0.98	0.72 (0.61–0.84)	90.6%
3. Delusion	0.8	0.63 (0.43–0.83)	96.9%
4. Insomnia	0.8	0.83 (0.75–0.92)	84.4%
5. Apathy	0.95	0.79 (0.68–0.9)	96.9%
6. Repeating	0.98	0.89 (0.8–0.98)	84.4%
7. Anxiety	0.93	0.65 (0.51–0.78)	93.8%
8. Depression	0.83	0.81 (0.7–0.92)	90.6%
9. Misidentification	0.85	0.93 (0.84–1)	96.9%
10. Hallucination	0.85	0.85 (0.75–0.94)	93.4%
11. Hoarding	0.9	0.85 (0.75–0.95)	93.8%
12. Rummaging	0.98	0.77 (0.67–0.88)	96.9%
13. Wandering	0.9	0.46 (0.24–0.67)	96.9%
14. Euphoria	0.9	0.54 (0.37–0.71)	93.8%

#### 3.3.2 Internal consistency

After pilot testing on 20 PwD and their paired CG, the BPSD‐T was adapted to 18 items due to the low internal consistency (<0.4) of two items. Then, the BPSD‐T was administrated to 168 CGs to assess its validity and reliability. The Kappa of inter‐rater reliability ranged between 0.63 and 0.93, except for euphoria (Kappa, 0.54) and wandering symptoms (Kappa, 0.46). Test–retest reliability was calculated as the overall percentage agreement, and it was higher than 84.4% for all items (Table 3). The average time to complete the NPI was 506 ± 196.86 s, compared with 230.89 ± 65.45 s for the BPSD‐T (*p* < .001). Relative to the other instruments, the BPSD‐T could assess the frequency of symptoms and CG distress in a shorter time (Supplementary Data S4).

#### 3.3.3 CFA

**Figure 1:**
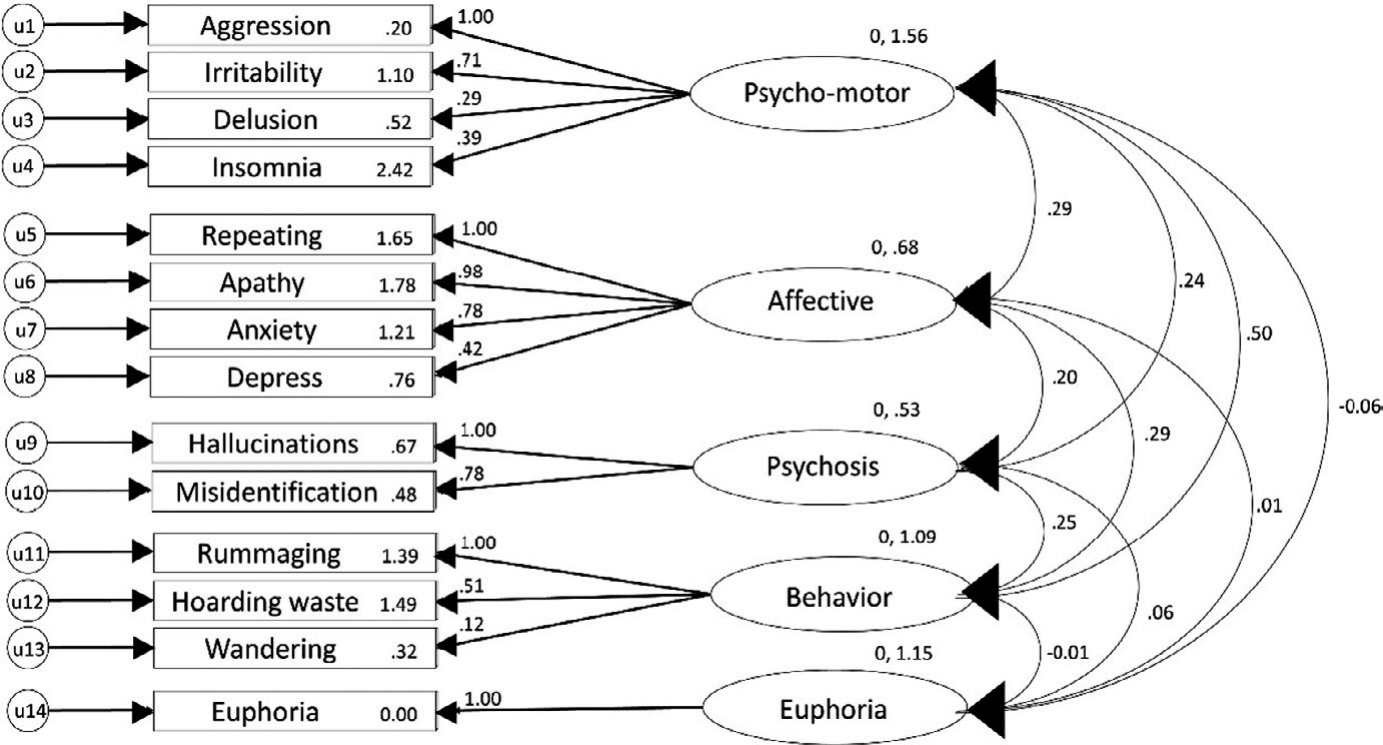
Standardized path coefficients for Model 7 (baseline data *n* = 168)

Before the factor analysis, three items with a frequency of less than 5% were excluded, as previously described in the statistics section. A total of 15 items from the BPSD‐T were analyzed in the CFA. The CFA for each model was calculated using the frequency score of each item of the BPSD‐T. Each CFA was performed using the ML estimation to explore the relationship between each BPSD‐T item. Table 4 summarizes the goodness‐of‐fit indices for the hypothesized models. Of the 9 models, Models 4, 5, and 6 met the marginal fit criteria. After removing the item of excessive sleep, resulting in 14 items remaining in the BPSD‐T, Model 7 met the goodness‐of‐fit criteria, with a CFI of 0.955, TLI of 0.940, SRMR of 0.058, and RMSEA of 0.031. Hence, the seventh model was selected as the definitive model for the latent structure of the BPSD‐T subscales. The standardized path coefficients for Model 7 are illustrated in Figure 1.

**Table 4:** Goodness‐of‐fit indices for the analyzed models

Model	*χ* ^2^	*df*	*p* value	*χ* ^2^/*df*	CFI	TLI	RMSEA	SRMR
Model 1	137.414	84	<.001	1.636	0.798	0.747	0.062	0.076
Model 2	185.295	89	<.001	2.082	0.635	0.569	0.080	0.090
Model 3	189.613	84	<.001	2.257	0.600	0.500	0.087	0.091
Model 4	113.337	84	.018	1.349	0.889	0.861	0.046	0.065
Model 5	98.868	68	.009	1.454	0.885	0.846	0.052	0.063
Model 6	105.810	77	.016	1.374	0.891	0.851	0.047	0.062
Model 7	78.916	68	.172	1.161	0.955	0.940	0.031	0.058
Model 8	160.716	82	<.001	1.960	0.702	0.618	0.076	0.089
Model 9	163.911	85	<.001	1.928	0.701	0.631	0.074	0.089

Abbreviations: CFI, Comparative Fit Index; RMSEA, Root‐Mean‐Square Error of Approximation; SRMR, Standardized Root‐Mean‐Square Residual; TLI, Tucker–Lewis Index.

#### 3.3.4 Convergent validity

**Figure 2:**
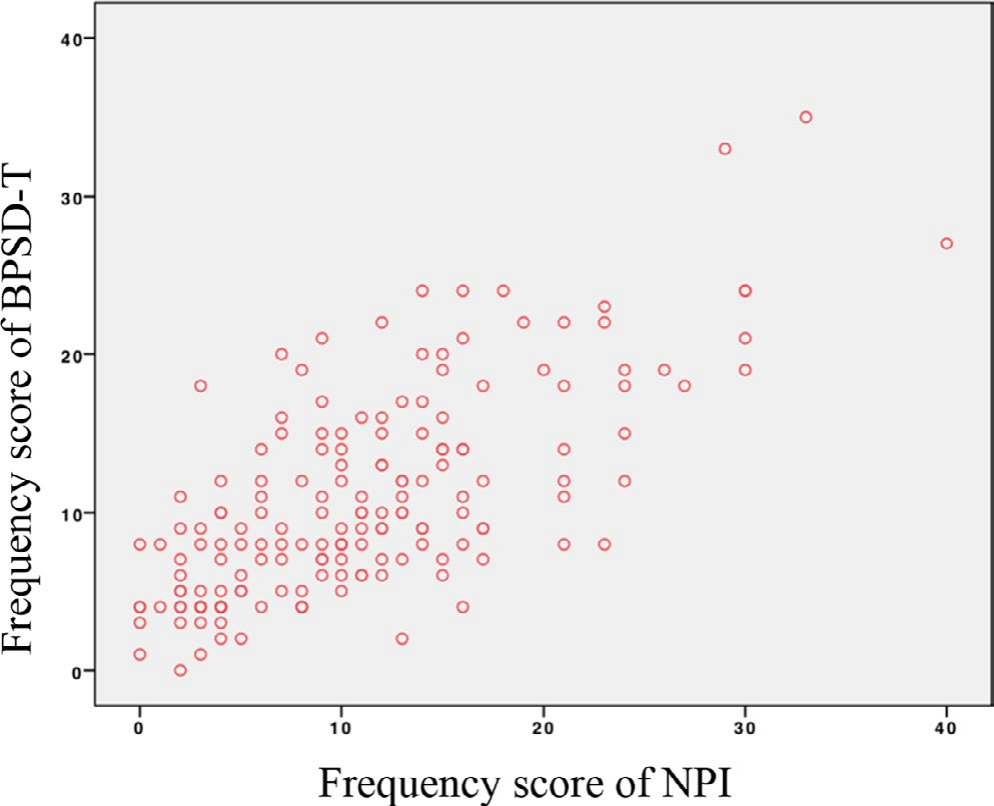
Correlation of frequency scores of NPI and BPSD‐T. Correlation between frequency scores of NPI and BPSD‐T: *r* = 0.661, *p* < .001

**Figure 3:**
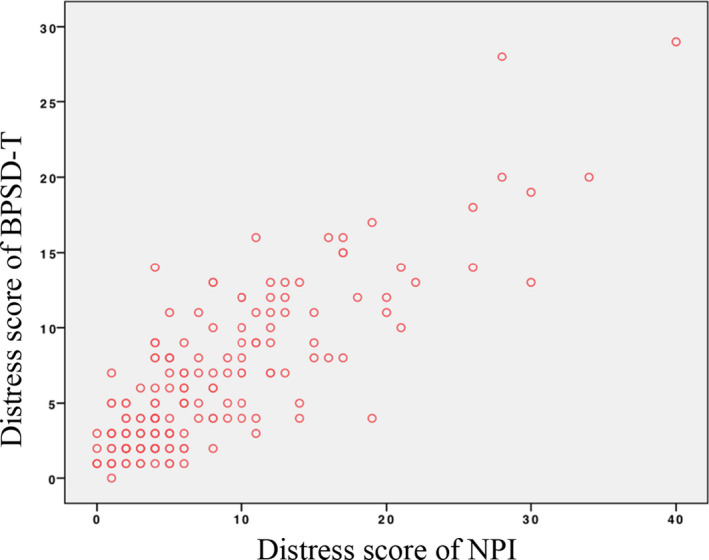
Correlation of distress scores of NPI and BPSD‐T. Correlation between distress scores of NPI and BPSD‐T: *r* = 0.758, *p* < .001

**Figure 4:**
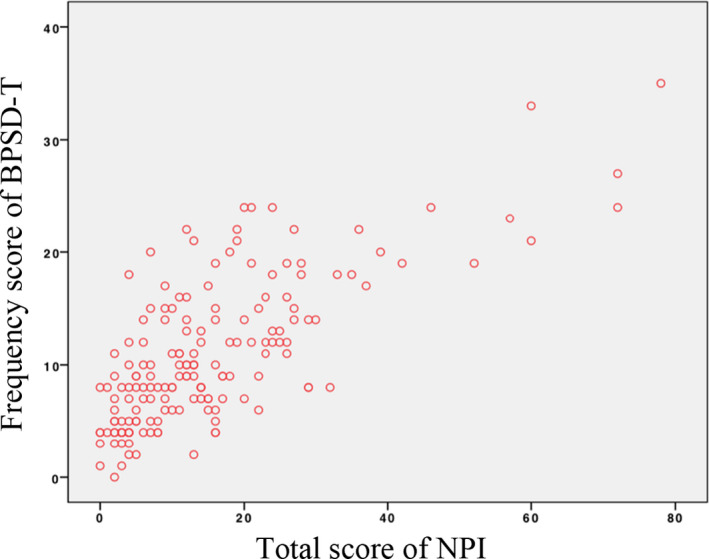
Correlation of frequency score of BPSD‐T and total score of NPI. Correlation between frequency score of BPSD‐T and total score of NPI: *r* = 0.684, *p* < .001

The correlation between the frequency score of BPSD‐T and the frequency score of NPI was *r* = 0.661 (*p* < .001; Figure 2). The CG distress subdomain score correlation coefficient was *r* = 0.758 (*p* < .001; Figure 3). The correlation between the frequency score of BPSD‐T and the total score of NPI was high (*r* = 0.684, *p* < .001; Figure 4).

## 4 DISCUSSION

The BPSD‐T is a more rapid and reliable test to evaluate BPSD from the common dementia types (AD, VaD, AD with SVD, and PDD) and severity of dementia. The development of this BPSD‐T was based on the most frequent and burdensome symptoms in the Thai population (Charernboon & Phanasathit, 2014; Graipaspong et al., 2016; Muangpaisan et al., 2010; Pinidbunjerdkool et al., 2014; Senanarong et al., 2005; Taemeeyapradit et al., 2014). It correlated well with the NPI in both the frequency score and the CG distress subscore, required less time to administer, and had high inter‐rater and test–retest reliabilities. Moreover, it could assess both the frequency and distress effects of the symptoms, which could not be done with the short version of NPI (NPI‐Q) despite their being equally time‐consuming tools (Kaufer et al., 2000). Our results suggest that the BPSD‐T could be used to evaluate BPSD and CG burden in the dementia population. Moreover, the BPSD‐T could be used to monitor BPSD progression during the follow‐up period. The BPSD‐T administration time was about 4 min, significantly less than the 8.5 min required by the NPI. In addition, the BPSD‐T was developed to be administered by general healthcare personnel. The BPSD‐T could be useful for evaluating BPSD in clinics with limited healthcare resources.

Combining groups of symptoms into clusters implies that the clustered symptoms should occur more frequently together. The hypothesis implies that the symptoms have a common neurobiological pathogenesis and shared neurotransmitters. In addition, some behavioral symptoms might be secondary to others. The CFA showed the goodness of fit of 5 factors of BPSD‐T, namely, a psychomotor syndrome (aggression, irritability, delusions, insomnia), an affective syndrome (apathy, repeating, anxiety, depression), a psychosis syndrome (misidentification, hallucinations), a behavior syndrome (hoarding, rummaging, wandering), and a euphoria syndrome (euphoria).

Depression and anxiety are usually found in the same factor in most studies (Aalten et al., 2008; Garre‐Olmo, López‐Pousa, Vilalta‐Franch, de Gracia Blanco, & Vilarrasa, 2010; Kang, Ahn, Kim, & Kim, 2010; Vaingankar et al., 2017), and apathy might be also in the same factor in a number of studies (Cheng, Kwok, & Lam, 2012; Frisoni et al., 1999; Johnson, Watts, Chapin, Anderson, & Burns, 2011). The different clusters of BPSD in each study might result from the natural course of BPSD, which change as the dementia progresses. The affective symptoms (depression, anxiety, and apathy) tend to emerge early in the disease process (van der Linde et al., 2016; Lyketsos et al., 2000). Therefore, they frequently emerge in the same cluster; this is consistent with the results of this study, in which depression, anxiety, and apathy were found in the same factor. As dementia progress, psychosis frequently coexists with agitation (Matsui et al., 2006). Delusion and hallucination usually co‐occur (Aalten et al., 2008; Cheng et al., 2012; Garre‐Olmo et al., 2010; Kang et al., 2010; Makimoto et al., 2019), and frequently combine with agitation/aggression and irritability (Feghali, Fares, & Abou Abbas, 2019; Frisoni et al., 1999; Johnson et al., 2011; Vaingankar et al., 2017). Our findings revealed that delusion and aggression/agitation are in the same factor, which is similar to the results of previous studies (Aarsland, Cummings, Yenner, & Miller, 1996; Lachs, Becker, Siegal, Miller, & Tinetti, 1992; Poletti et al., 2013). This finding might imply that agitation/aggression is secondary to delusions. Previous studies consistently found that some BPSD are loaded on more than one factor. The exclusion of these BPSD items from the factorial solution significantly improved the model fitting. Euphoria and aberrant motor behavior are examples of that (Aalten et al., 2008; Cheng et al., 2012; Feghali et al., 2019; Poletti et al., 2013). In our study, excluding “excessive sleep” item improved the model fit.

The prevalence of BPSD was observed to be high. This is consistent with previous studies in specialized clinics, where the diagnosis of dementia was already present (Charernboon & Phanasathit, 2014; Garre‐Olmo et al., 2010; Petrovic et al., 2007; Taemeeyapradit et al., 2014). This is unlike studies that recruited participants from community‐based surveys, where the prevalence of BPSD was usually much lower (Haibo et al., 2013; Lyketsos et al., 2000; Vaingankar et al., 2017). The two most common BPSD were nighttime behavior and aberrant motor behavior, which are similar to the results of one study conducted at a memory clinic (Charernboon & Phanasathit, 2014). However, the two most common BPSD in other studies were typically apathy and depression (Frisoni et al., 1999; Garre‐Olmo et al., 2010; Kang et al., 2010; Lyketsos et al., 2000; Petrovic et al., 2007; Poletti et al., 2013). Irritability and agitation were also common BPSD in many studies. Similar to other studies, euphoria was the rarest BPSD (Cheng et al., 2012; Kang et al., 2010; Poletti et al., 2013). Several factors have been proposed to cause the different prevalences and clustering of BPSD symptoms, including the fluctuating course of BPSD, dementia severity, type of dementia, and external influences such as the caregiving pattern, psychotropic and antidementia drug used, social context, and understanding of the questionnaire (Canevelli et al., 2013; Johnson et al., 2011).

The strengths of the study include the following. First, there was high participant response rate (86.7%) after using the systematic sampling method; this meant that the studied population was representative of the target population. Second, the study included the common subtypes and all severity levels of dementia, as categorized by the Global CDR scale. Third, the finding that changes in the BPSD‐T score were correlated with the severity of dementia suggests that the BPSD‐T could be used during the follow‐up period to examine the progression of the disease. Fourth, the authors developed the tool using local data to reflect the specific cultural and ethnic context of Thailand. Moreover, recent studies have reported similar BPSD characteristics for Asian PwD, confirming that the types of symptoms included in the BPSD could be the same in all Asian countries. This therefore suggests that the BPSD‐T might be useful in other Asian countries (Haibo et al., 2013; Makimoto et al., 2019).

This study has some limitations. As it was conducted at a large urban university hospital, the baseline characteristics of the subjects may have been different from other areas of the country. For example, we observed a high educational level among the CGs (69.6% were educated to at least a bachelor's degree level); this may have resulted in a higher rate of reported BPSD (Cerejeira, Lagarto, & Mukaetova‐Ladinska, 2012) and a lower rate of CG burden than for CGs with lower levels of education (Sink, Covinsky, Barnes, Newcomer, & Yaffe, 2006). However, the higher‐educated CGs might have been more accurate in reporting the BPSD due to their better understanding of the study questionnaires. More research is needed to evaluate the validity of the test in the community, in rural areas, and in the primary care setting.

## 5 CONCLUSIONS

The BPSD‐T is a quick, reliable, and valid test to evaluate BPSD from common dementia subtypes and all severities. It has a good correlation with the NPI in terms of total score and CG burden. It can be also be performed by nonphysician healthcare personnel. All of these positive findings support its use in routine clinical practice for the recognition of BPSD in order to improve the quality of patient care and to reduce the CG burden.

## CONFLICT OF INTEREST

The authors have no conflicts of interest related to this study to disclose.

## AUTHOR CONTRIBUTIONS

Phannarus H and Muangpaisan W involved in conceptualization, methodology, writing of original draft, and visualization. Phannarus H, Muangpaisan W, and Supapueng O involved in software, validation, and formal analysis. All coauthors involved in investigation, resources, data curation, and writing of final paper. Muangpaisan W involved in project administration and supervision.

### Peer Review

The peer review history for this article is available at https://publons.com/publon/10.1002/brb3.1816.

## Supporting information

Supplementary material

## Data Availability

The data that support the findings of this study are available from the corresponding author upon reasonable request.
