# Supplementary material for: Development of a Thai tool for assessing behavioral and psychological symptoms of dementia: A confirmatory factor analysis
Source: Brain Behav. 2020 Aug 28;10(11):e01816. doi: 10.1002/brb3.1816 (PMC7667320; doi:10.1002/brb3.1816)
Supplement: Supplementary file 1 — Supplementary material [file BRB3-10-e01816-s001.docx]

**Supplementary Data of “Development of a Thai Tool for Assessing Behavioral and Psychological Symptoms of Dementia: A Confirmatory Factor Analysis”**

**Supplementary Data S1**

**Behavioral and Psychological Symptoms of Dementia (BPSD-T)**

Instruction:

Rate the absence/presence, frequency, and distress effects of BPSD symptoms over the last month.

Scale of frequency: Scale of distress which affects caregivers:

1 = below 2 times/month 1 = no effect

2 = 2–3 times/month 2 = little effect on caregivers

3 = weekly, at least 4 times/month 3 = some effects on caregivers, still bearable

4 = almost every day 4 = a lot of effect on caregivers which cannot be handled

| Items | Absent/  Present | Frequency | Distress effect |
| --- | --- | --- | --- |
| 1. Does the patient have verbal or physical aggression? |  |  |  |
| 1. Does the patient get easily irritated? |  |  |  |
| 1. Does the patient believe that people are trying to harm him/her or steal something from him/her (which you know is not true)? |  |  |  |
| 1. Does the patient have difficulty sleeping? |  |  |  |
| 1. Does he/she collect wasted stuff or any garbage? |  |  |  |
| 1. Does the patient repeatedly ask/say something, or do an activity again and again? |  |  |  |
| 1. Does the patient have unreasonably anxious feelings about everyday events? |  |  |  |
| 1. Does the patient believe that the people on TV are real people? |  |  |  |
| 1. Does the patient feel worthless or sad, cry without reason, feel bored or discouraged, or express a wish to die? |  |  |  |
| 1. Does the patient describe hearing—or act as if he/she hears—imaginary voices, or see things that are not actually present? |  |  |  |
| 1. Has the patient lost interest in, or seem to be indifferent about, his/her surroundings? |  |  |  |
| 1. Does the patient rummage through stuff or hide some things from others? |  |  |  |
| 1. Does he/she walk aimlessly? |  |  |  |
| 1. Does the patient appear to be inappropriately cheerful or happy at times? |  |  |  |

**Supplementary Data S2**

**Models for the confirmatory factor analysis**

**Model 1**, a four-factor model, comprised hyperactivity (euphoria, irritability, aggression, wandering, hoarding, rummaging); psychosis (delusion, hallucinations, insomnia, misidentification); affective symptoms (depression, anxiety, repeating); and apathy (apathy, excessive sleep).

**Model 2**, a four-factor model, comprised psychosis/behavior (delusion, hallucination, aggression, apathy, irritability, insomnia, misidentification, hoarding, rummaging, wandering); and mood/restlessness (depression, anxiety, euphoria, repeating, excessive sleep).

**Model 3**, a four-factor model, comprised behavior (repeating, aggression, rummaging, wandering, hoarding); psychosis (hallucinations, delusion, misidentification); mood (depression, anxiety, irritability, apathy, euphoria); and sleep (insomnia, excessive sleep).

**Model 4**, a five-factor model, comprised psychomotor symptoms (aggression, irritability, delusion, insomnia); affective symptoms (apathy, repeating, anxiety, depression); psychosis (misidentification, hallucination); behavior (hoarding, rummaging, wandering); and euphoria (excessive sleep, euphoria).

**Model 5,** a five-factor model, comprised psychomotor symptoms (aggression, irritability, delusion, insomnia); affective symptoms (apathy, repeating, anxiety, depression); psychosis (misidentification, hallucination); behavior (hoarding, rummaging, wandering); and excessive sleep (excessive sleep).

**Model 6,** a six-factor model, comprised psychomotor symptoms (aggression, irritability, delusion, insomnia); affective symptoms (apathy, repeating, anxiety, depression); psychosis (misidentification, hallucination); behavior (hoarding, rummaging, wandering); excessive sleep (excessive sleep); euphoria (euphoria)

**Model 7,** a five-factor model, comprised psychomotor symptoms (aggression, irritability, delusion, insomnia); affective symptoms (apathy, repeating, anxiety, depression); psychosis (misidentification, hallucination); behavior (hoarding, rummaging, wandering); and euphoria ( euphoria).

**Model 8**, a five-factor model, comprised behavior (aggression, irritability, hoarding, rummaging, wandering); psychosis (delusion, hallucination, misidentification); mood (depression, apathy, insomnia, anxiety, repeating); euphoria; and excessive sleep.

**Model 9**, a four-factor model, comprised behavior (aggression, irritability, hoarding, rummaging, wandering); psychosis (delusion, hallucination, misidentification); mood (depression, apathy, insomnia, anxiety, repeating, excessive sleep); and euphoria.

**Supplementary Data S3**

Baseline characteristics of the caregivers (n = 168)

| Caregivers | |
| --- | --- |
| Age (years), mean ± SD | 55.9 ± 13.4 |
| Woman, n (%) | 136 (81) |
| Marital status, n (%)  Married  Single | 101 (60.1)  61 (36.3) |
| Education, n (%)  < Bachelor’s degree  ≥ Bachelor’s degree | 51 (30.4)  117 (69.6) |
| Relationship to PwD, n (%)  Child  Spouse  Formal caregiver | 105 (62.5)  30 (17.9)  11 (6.5) |
| Duration of contact/day, n (%)  > 12 hours  4–12 hours | 108 (64.3)  58 (34.5) |
| Duration of being a caregiver, n (%)  > 4 years  1–4 years | 81 (48.2)  79 (47) |

Abbreviations: PwD, people with dementia; SD, standard deviation

**Supplementary Data S4**

Characteristics of the instruments

|  | BPSD-T | BEHAVE-AD | NPI | NPI-Q | ABS | CGA-NPI | CUSPAD | BRSD | DBDI | COBRA scale |
| --- | --- | --- | --- | --- | --- | --- | --- | --- | --- | --- |
| Items | 14 | 25 | 12 | 12 | 10 | 12 | 18 | 46 | 72 | 30 |
| Frequency of symptoms | + | - | + | - | + | + | + | + | + | + |
| Severity of symptoms | - | + | + | + | - | + | + | + | + | + |
| Caregiver distress score | + | - | + | + | - | + | - | - | - | - |
| Time to complete, mean (minutes) | 4 | 20 | 15–20 | 5 | 1 | 21 | 10–25 | - | 15 | - |

**Abbreviations:**

ABS, Abe’s Behavioral and Psychological Symptoms in Dementia Score (Abe et al., 2015);

BEHAVE-AD, Behavioral Pathology in Alzheimer’s Disease Rating Scale (Reisberg et al., 1996);

BPSD-T, Thai tool for Assessing Behavioral and Psychological Symptoms of Dementia;

BRSD, Behavior Rating Scale for Dementia (Mack et al., 1999);

CGA-NPI, Caregiver-Administered Neuropsychiatric Inventory (Kang et al., 2004);

COBRA, Caretaker Obstreperous-Behavior Rating Assessment Scale (Drachman et al., 1992);

CUSPAD, Columbia University Scale for Psychopathology in Alzheimer’s Disease (Devanand et al., 1992);

DBDI, Dementia Behavior Disturbance Inventory (Tang et al., 2006);

NPI, Neuropsychiatric Inventory(Cummings, 1997);

NPI-Q, Neuropsychiatric Inventory Questionnaire (Kaufer et al., 2000)

**Reference**:

Abe, K., Yamashita, T., Hishikawa, N., Ohta, Y., Deguchi, K., Sato, K., Matsuzono, K., Nakano, Y., Ikeda, Y., Wakutani, Y., & Takao, Y. (2015). A new simple score (ABS) for assessing behavioral and psychological symptoms of dementia. *Journal of the Neurological Sciences*, *350*(1–2), 14–17. https://doi.org/10.1016/j.jns.2015.01.029

Cummings, J. L. (1997). The Neuropsychiatric Inventory: assessing psychopathology in dementia patients. *Neurology*, *48*(5 Suppl 6), S10-6. https://doi.org/10.1212/wnl.48.5_suppl_6.10s

Devanand, D. P., Miller, L., Richards, M., Marder, K., Bell, K., Mayeux, R., & Stern, Y. (1992). The Columbia University Scale for Psychopathology in Alzheimer’s disease. *Archives of Neurology*, *49*(4), 371–376. https://doi.org/10.1001/archneur.1992.00530280051022

Drachman, D. A., Swearer, J. M., O’Donnell, B. F., Mitchell, A. L., & Maloon, A. (1992). The Caretaker Obstreperous-Behavior Rating Assessment (COBRA) Scale. *Journal of the American Geriatrics Society*, *40*(5), 463–470. https://doi.org/10.1111/j.1532-5415.1992.tb02012.x

Kang, S. J., Choi, S. H., Lee, B. H., Jeong, Y., Hahm, D. S., Han, I. W., Cummings, J. L., & Na, D. L. (2004). Caregiver-Administered Neuropsychiatric Inventory (CGA-NPI). *Journal of Geriatric Psychiatry and Neurology*, *17*(1), 32–35. https://doi.org/10.1177/089198873258818

Kaufer, D. I., Cummings, J. L., Ketchel, P., Smith, V., MacMillan, A., Shelley, T., Lopez, O. L., & DeKosky, S. T. (2000). Validation of the NPI-Q, a brief clinical form of the Neuropsychiatric Inventory. *The Journal of Neuropsychiatry and Clinical Neurosciences*, *12*(2), 233–239. https://doi.org/10.1176/jnp.12.2.233

Mack, J. L., Patterson, M. B., & Tariot, P. N. (1999). Behavior Rating Scale for Dementia: development of test scales and presentation of data for 555 individuals with Alzheimer’s disease. *Journal of Geriatric Psychiatry and Neurology*, *12*(4), 211–223. https://doi.org/10.1177/089198879901200408

Reisberg, B., Auer, S. R., & Monteiro, I. M. (1996). Behavioral pathology in Alzheimer’s disease (BEHAVE-AD) rating scale. *International Psychogeriatrics*, *8 Suppl 3*, 301–304. https://doi.org/10.1097/00019442-199911001-00147

Tang, L.-Y., Yip, P.-K., Wu, E.-C., & Leung, K.-K. (2006). The development and validation of a dementia behavior disturbance inventory. *International Psychogeriatrics*, *18*(1), 95–110. https://doi.org/10.1017/S1041610205002164
